# Multiorgan Dysfunction in a Patient With Adult-Onset Still’s Disease Flare: A Case Report

**DOI:** 10.7759/cureus.60400

**Published:** 2024-05-16

**Authors:** Akhila Arya P V, Deepak R Vangipuram, Madhumita Rondla, Dileep Unnikrishnan

**Affiliations:** 1 Internal Medicine, Bridgeport Hospital, Stratford, USA; 2 Internal Medicine, Bridgeport Hospital, Bridgeport, USA; 3 Internal Medicine, Texas Tech University Health Sciences Center El Paso, El Paso, USA; 4 Internal Medicine, Cloudphysician Healthcare, Long Branch, USA; 5 Internal Medicine, Monmouth Medical Center, Long Branch, USA

**Keywords:** adult-onset still’s disease (aosd), multiorgan dysfunction, acute tubular injury, ischemic hepatitis, fulminant myocarditis

## Abstract

Adult-onset Still’s disease (AOSD) is a rare multisystem inflammatory disorder. A 71-year-old lady who was on treatment for AOSD presented with clinical evidence of heart failure and was subsequently found to have impaired renal and hepatic function. Following extensive workup including a liver biopsy, the cause of liver dysfunction was determined to be congestive hepatopathy, while renal dysfunction was presumed to stem from the low output state. The etiology of myocardial dysfunction, driving liver and kidney injury, was considered to be myocarditis from AOSD or global myocardial dysfunction from a systemic inflammatory state. Management involved pulse-dose glucocorticoids followed by taper and anakinra for AOSD, alongside goal-directed medical therapy for cardiac failure. At follow-up after a month, hepatic and renal function had fully recovered, whereas cardiac function remained compromised, evidenced by persistently depressed ejection fraction and global hypokinesia on a repeat echocardiogram. This report delineates a systematic approach to multiorgan dysfunction in a patient with a rare condition such as AOSD and reviews the reported causes of hepatic and cardiac involvement in AOSD.

## Introduction

Adult-onset Still’s disease (AOSD) is a multisystem inflammatory disorder with a prevalence of 0.16-0.6 per 100,000 population [1]. Due to the rarity of this disease, there is a notable paucity of data regarding its long-term progression and the prognosis of significant organ involvement. In the heart, AOSD may affect the pericardium, myocardium, or endocardium [2]. The etiology of liver involvement is multifaceted, with manifestations ranging from asymptomatic elevations in liver enzymes to acute hepatic failure. This report details the case of a patient with AOSD undergoing treatment who developed new-onset heart failure concurrent with hepatic and renal injury. We delineate our diagnostic approach to what initially presented as a complex case and discuss the potential etiologies of cardiac and hepatic involvement in AOSD, with a particular emphasis on myocarditis.

## Case presentation

A 71-year-old lady, presented with dyspnea, orthopnea, and bilateral leg swelling progressively worsening over the last two weeks. She also experienced right upper quadrant abdominal pain associated with nausea and vomiting. Additionally, she reported malaise, joint pains, and a pink skin rash over her chest. She was diagnosed with AOSD five years ago (as per Yamaguchi criteria) when she presented with fever, rash, and arthralgia and was being treated with anakinra, methotrexate, hydroxychloroquine, and prednisone [3]. However, following multiple hospitalizations for infections including COVID-19 and streptococcal toxic shock syndrome in the past two months before the current presentation, her medication regimen was changed to hydroxychloroquine with prednisone, on which she was doing well.

On examination, her heart rate was 64 beats/minute, blood pressure was 102/91 mmHg, temperature was 97.3°F, and SpO_2_ was 97% on room air. Physical examination revealed bilateral grade 2 pitting pedal edema, a faint pink maculopapular rash on her chest, mild swelling and tenderness of bilateral wrists and ankles without warmth or erythema, but there was no asterixis or elevated jugular venous pressure. Chest examination showed normal breath sounds bilaterally with no inspiratory crackles or wheezing, and cardiac auscultation revealed no murmurs or added sounds. The abdomen was not distended and there was no clinical evidence of ascites. Mild tenderness was noted in the right upper quadrant.

Investigations and differential diagnoses

Laboratory parameters at admission are summarized in Table 1. Notably, there was chronic anemia with a hemoglobin level of 11.3 g/dL, which was her baseline (normal range = 11.7-15.5 g/dL). Liver function tests revealed significantly elevated levels of alanine aminotransferase (ALT) at 1,754 U/L (normal range = 10-35 U/L), aspartate aminotransferase (AST) at 1,278 U/L (normal range = 10-35 U/L), and alkaline phosphatase (ALP) at 369 U/L (normal range = 37-153 U/L), along with an international normalized ratio of 2.26. Additionally, inflammatory markers were elevated, with C-reactive protein (CRP) at 118 mg/L, erythrocyte sedimentation rate (ESR) at 21 mm/hour, and lactate dehydrogenase (LDH) at 4,169 U/L. Serum ferritin levels were markedly elevated at 3,995 ng/mL. The patient also had an elevation of creatinine to 1.56 mg/dL from a baseline of 1.1 mg/dL, suggesting acute kidney injury. Urine analysis was without sediments, and the urine protein creatinine ratio was 0.37 mg/mg.

**Table 1:** Laboratory parameters at admission.

Parameter	Patient’s value	Reference range
Hemoglobin	11.3 g/dL	11.7–15.5 g/dL
Platelets	221 × 1,000/µL	150–420 × 1,000/µL
White cell count	10.6 × 1,000/µL	4.0–11.0 × 1,000/µL
Neutrophils	92.1%	39.0–72.0%
Lymphocytes	3.4%	17.0–50.0%
Sodium	135 mmol/L	136–144 mmol/L
Potassium	5.1 mmol/L	3.3–5.3 mmol/L
Blood urea nitrogen	66 mg/dL	6–20 mg/dL
Creatinine	1.56 mg/dL	0.40–1.30 mg/dL
Total protein	6.5 g/dL	6.6–8.7 g/dL
Albumin	3.5 g/dL	3.6–4.9 g/dL
Total bilirubin	0.9 mg/dL	Up to 1.2 mg/dL
Alanine aminotransferase	1,754 U/L	10–35 U/L
Aspartate aminotransferase	1,278 U/L	10–35 U/L
Alkaline phosphatase	369 U/L	37–153 U/L
High-sensitivity troponin T	18 ng/L	<14 ng/L
N-terminal prohormone of brain natriuretic peptide	36,632 pg/mL	<125 pg/mL
Procalcitonin	0.19 ng/mL	<0.25 ng/mL
Antinuclear antibodies	<1:80	<1:80
C3	65 mg/dL	90–180 mg/dL
C4	11 mg/dL	10–40 mg/dL
Anti-liver-kidney microsomal antibody	<2.0 U	<20.0 U
Anti-smooth muscle antibody	6 U	<20.0 U
Immunoglobulin G	1,095 mg/dL	700–1,600 mg/dL
Immunoglobulin M	230 mg/dL	40–230 mg/dL
Immunoglobulin A	258 mg/dL	70–470 mg/dL
Alpha-1-antitrypsin	228 mg/dL	90–200 mg/dL
Ferritin	3,995 ng/mL	13–150 ng/mL
Triglycerides	323 mg/dL	<150 mg/dL
Lactate dehydrogenase	4,169 U/L	122–241 U/L
C-reactive protein	118 mg/L	<10.0 mg/L
Erythrocyte sedimentation rate	21 mm/hour	0–20 mm/hour

**Figure 1:**
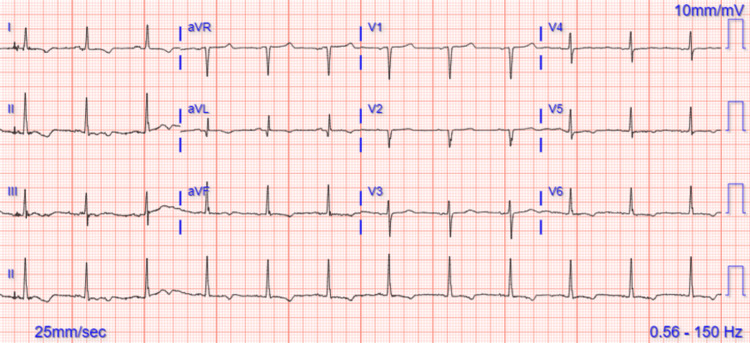
Electrocardiogram showing sinus rhythm, T inversions in leads I, avL, V5, V6, II, III, and aVF.

This hepatocellular pattern of liver enzyme elevation prompted consideration of potential causes, including alcoholic hepatitis, viral hepatitis, AOSD flare, drug-induced liver injury (DILI) from recent antibiotic use, autoimmune hepatitis, macrophage activation syndrome, ischemic hepatitis, congestive hepatopathy, sepsis, genetic hepatic diseases (Wilson’s disease, hemochromatosis), toxins, and malignancy. Further testing was directed against ruling in or out the above differentials. The viral panel, autoimmune hepatitis panel, and toxicology screen were negative, and serum ceruloplasmin and alpha-1 antitrypsin were within normal limits. Alcoholic hepatitis was ruled out given no history of alcohol use and negative serum ethanol levels. Both blood and urine cultures were sterile. Hepatitis and infectious disease workups are summarized in Table 2 and Table 3, respectively. Portal vein thrombosis was ruled out with duplex ultrasound and there was no concern for malignancy on abdominal imaging. Even though the patient had an elevation of serum ferritin and CRP and mild anemia, she did not meet the criteria for hemophagocytic lymphohistiocytosis [4]. DILI was lower in the differentials due to low clinical suspicion. Clinical evidence of congestive heart failure and elevated N-terminal prohormone of brain natriuretic peptide at 36,632 pg/mL (normal range = <125 pg/mL) indicated cardiac involvement. An echocardiogram confirmed severe global hypokinesis of the left ventricle, with an ejection fraction (EF) of 20% and evidence of right ventricular dysfunction. There was only a mild elevation of troponin T to 18 ng/L without any serial rise (normal range = <14 ng/L) and electrocardiography (ECG) revealed slight T-wave inversion in the inferior and lateral leads (see Figure 1) which persisted in subsequent ECGs.

**Table 2:** Hepatitis workup.

Parameter	Result	Normal values
Toxicology screening (serum)	Negative	
Acetaminophen level	<5 µg/mL	10–30 µg/mL
Antinuclear antibody screen	<1:80	<1:80
Liver-kidney microsome antibody, IgG	<2.0 U	<20 U
Smooth muscle (F-Actin) antibody, IgG w/reflex to titer	6 U	<20 U
Immunoglobulin G	1,259 mg/dL	700–1,600 mg/dL
Ceruloplasmin	38 mg/dL	18–51 mg/dL
Ferritin	3,995 ng/mL	13–150 ng/mL
Iron saturation	17%	15–50%
Alpha-1-antitrypsin	228 mg/dL	90–200 mg/dL

**Table 3:** Infectious disease workup. HbsAg = hepatitis B surface antigen; VCA = viral capsid antigen; PCR = polymerase chain reaction

Parameter	Result
Blood cultures	No growth after 5 days of incubation
Urine culture	No growth after 5 days of incubation
Hepatitis A antibody	Negative
Hepatitis B (HbsAg; IgM core antibody)	Negative
Hepatitis C antibody	Negative
Hepatitis E antibody	Negative
Human immunodeficiency virus 1 and 2	Negative
Epstein-Barr virus VCA IgM	Negative
Cytomegalovirus quantitative PCR	Not detected
Herpes simplex virus 1 and 2 PCR	Not detected
SARS-CoV-2, influenza, respiratory syncytial virus	Negative

Likely differentials were narrowed down to hepatic dysfunction from AOSD flare (recent change in immunosuppressive regimen, elevated CRP, and ferritin) or congestive hepatopathy from heart failure (clinical presentation of heart failure and depressed left ventricular function on echocardiogram). As severe hepatic dysfunction is unlikely in AOSD flare in the absence of macrophage activation syndrome, we proceeded with a liver biopsy on day five of the hospital stay to establish a definitive diagnosis. It showed centrilobular and pan-lobular necrosis with focal ischemic cholangiopathy, which was consistent with ischemic injury. There was no evidence of autoimmune hepatitis. Thus, it was concluded that the low cardiac output state and systemic vascular congestion were the primary contributors to liver dysfunction and possibly acute kidney injury.

The potential etiologies of myocardial dysfunction considered included ischemia, myocarditis due to AOSD, or global myocardial dysfunction in the setting of systemic inflammation. The echocardiogram demonstrated global hypokinesia of the left ventricle with an EF of 20%, without typical patterns suggestive of stress cardiomyopathy. The absence of significant troponin elevation, minimal ECG changes (only slight T-wave inversions), or regional wall motion abnormalities on echocardiogram argued against an ischemic etiology. COVID-19-induced myocarditis was considered but thought to be unlikely given onset after two months.

Treatment, outcome, and follow-up

**Figure 2:**
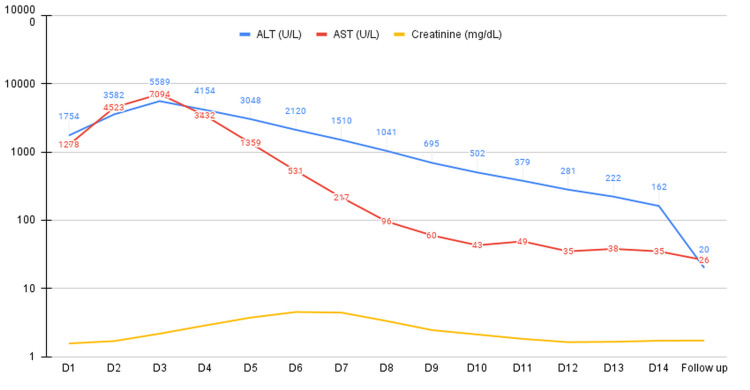
Trends of liver enzymes (AST, ALT, and creatinine) during hospital stay. Steroids were initiated on day three. AST = aspartate aminotransferase; ALT = alanine transaminase

The primary treatment consisted of methylprednisolone 1 g intravenously once a day for three days, followed by oral dexamethasone. Adjunctive therapies included intravenous furosemide for diuresis and lactulose to prevent hepatic encephalopathy. Clinical status and laboratory parameters began to improve by the third day of methylprednisolone initiation. At the time of discharge at the end of two weeks, ALT and AST levels improved to 162 U/L and 35 U/L, respectively. Creatinine was noted to be at 1.71 mg/dL, CRP at 19.7 mg/L, and ESR at 12 mm/hour (Figure 2). Carvedilol was subsequently added, and anakinra was resumed upon discharge.

At the first follow-up visit three weeks later, liver enzymes were within normal limits, and creatinine levels remained above baseline at 1.72 mg/dL. Cardiac function did not exhibit significant improvement, with the EF persisting at 25-30% and global left ventricular hypokinesia. Guideline-directed medical therapy for heart failure was initiated. Subsequent outpatient evaluation for cardiac ischemia via nuclear medicine myocardial perfusion single-photon emission computed tomography (stress and rest) revealed mildly depressed global left ventricular systolic dysfunction and mild global hypokinesis but no evidence of ischemia. At the nine-month follow-up, there was a recovery of EF to 60% and ECG changes returned to normal.

## Discussion

Still’s disease is a multisystem inflammatory condition clinically characterized by fever, skin rash, and polyarthritis. Hepatosplenomegaly, lymphadenopathy, and serositis are commonly observed. Originally described in the pediatric population in 1896 by George Still as juvenile idiopathic arthritis, the term was expanded to “adult-onset Still’s disease” in the 1970s to describe a similar presentation in people aged more than 16 years [5].

The prevalence of this disease is low, 0.16- 0.6 per 100,000 people, which is a limitation of its study [1]. Classically, a bi-modal age distribution has been described, with the first peak in the age group of 15-25 years, and a second peak at 36-46 years. A gender predisposition is not clear, as studies are limited and regional [6]. While the exact etiology of AOSD is unclear, genetic and infectious triggers have been postulated. Subsequent steps in pathogenesis include macrophage activation, a cytokine storm, neutrophil activation, and neutrophil extracellular trap release. The lack of adequate immune regulation due to reduced transforming growth factor-beta and T-reg cells perpetuates a cytokine storm. Of note, high levels of serum ferritin are observed in AOSD and correlated with disease severity [7].

The characteristic clinical features include fever spikes, an evanescent maculopapular rash, polyarthralgia, leukocytosis (>10,000/mm^3^), and elevated serum ferritin levels. Systemic inflammation may manifest as myalgia, myositis, pericarditis, myocarditis, pleuritis, hepatitis, splenomegaly, and lymphadenopathy. Laboratory findings typically show neutrophilic leukocytosis, elevated ESR and CRP, as well as liver enzyme elevation. Notably, antinuclear antibodies and rheumatoid factor are usually negative. The disease course may be monophasic (a single episode followed by resolution), intermittent (recurrent flares with remission between episodes), or chronic (persistently active disease). While the monophasic type is characterized by systemic symptoms, the chronic type typically presents with joint-related symptoms [8].

For research purposes, several diagnostic criteria have been proposed, with the most widely accepted ones being the Yamaguchi criteria and Fautrel’s criteria [3,9]. It is important to consider various differential diagnoses alongside Still’s disease, including viral infections, lymphoproliferative disorders, and other rheumatological conditions such as rheumatoid arthritis, systemic lupus erythematosus, vasculitis, and autoinflammatory conditions such as periodic fever syndromes.

We report the case of a patient with AOSD who presented with multiple organ dysfunction, including the heart, liver, and kidneys, and describe the systematic diagnostic process employed.

**Figure 3:**
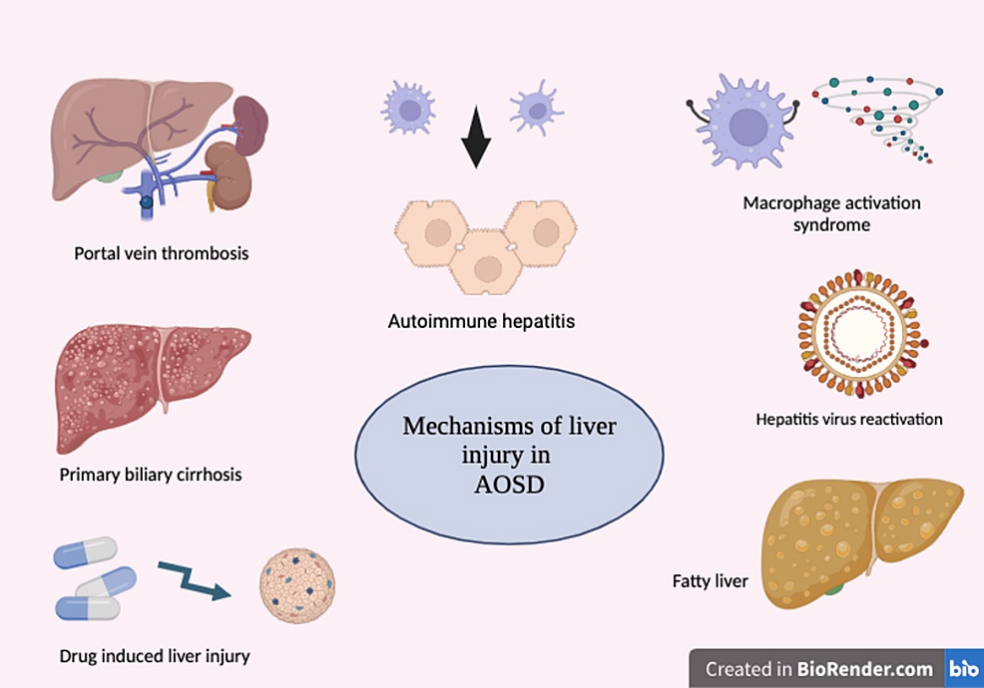
Depiction of mechanisms of liver injury in AOSD. Created with BioRender.com. AOSD = adult-onset Still’s disease

Hepatic involvement in AOSD can vary from asymptomatic mild elevations in liver enzymes to fulminant liver failure. Figure 3 summarizes some previously reported causes of liver dysfunction in AOSD. These include autoimmune hepatitis, macrophage activation syndrome, portal vein thrombosis, primary biliary cirrhosis, hepatitis virus reactivation, fatty liver, and DILI. It is crucial to note that liver dysfunction should not be automatically attributed to a disease flare without first ruling out other potential causes.

Cardiac involvement in AOSD can be in the form of pericarditis, effusion, pericardial effusion, pericardial tamponade, myocarditis, or non-infective endocarditis [2]. Myocarditis in Still’s disease is considered a rare entity, with fewer than 50 cases reported in the literature. It has been categorized into the following three groups: (1) possible myocarditis, characterized by elevated levels of cardiac biomarkers, abnormal ECG findings, or abnormal cardiac function on echocardiogram/cardiac MRI; (2) probable myocarditis, which includes the criteria for possible myocarditis along with additional cardiovascular symptoms; and (3) definite myocarditis, which requires confirmation via cardiac biopsy [10]. In a study by Smith et al., troponin elevation was reported in only 34% of patients with biopsy-proven myocarditis [11]. Additionally, a review by Gerfaud-Valentin et al. examined 24 cases of myocarditis in AOSD, where the clinical presentation resembled that of non-AOSD myocarditis but included markers of systemic inflammation. Interestingly, ECG findings were normal in 29% of patients, and half of the patients presented with myocarditis at the onset of the disease [12]. Another potential mechanism for myocardial dysfunction during an AOSD flare is non-specific myocardial involvement within the context of a severe systemic inflammatory response. This non-specific myocardial dysfunction is attributed to the myocardial depressant properties of inflammatory molecules, including cytokines, similar to sepsis-induced cardiomyopathy [13]. This hypothesis is supported by the elevated mean values of inflammatory markers such as ESR, CRP, and ferritin in 48 cases of myocarditis in AOSD analyzed by Gracia-Ramos et al. [14].

The treatment approach for myocarditis in AOSD consists of two arms, namely, disease-specific and non-disease-specific. Disease-specific treatment involves immunosuppression, while non-specific treatment includes the use of diuretics, beta-blockers, renin-angiotensin-aldosterone system inhibitors, etc., similar to the management of other types of heart failure [12]. Corticosteroids are the first-line specific therapy, with treatment response reported in up to 60% of patients. Disease-modifying anti-rheumatic drugs, typically methotrexate, are considered second-line therapy. Additionally, other treatment options include IL-1 antagonists (such as anakinra, canakinumab, rilonacept), IL-6 antagonists (tocilizumab), and tumor necrosis factor (TNF) inhibitors (infliximab, etanercept, adalimumab) [15]. It is critical to note that TNF inhibitors are contraindicated in the context of heart failure. Information regarding prognosis is derived mainly from case series. Mortality is low, but information about the recovery of cardiac function or long-term outcomes is unknown [12].

## Conclusions

AOSD is a multiorgan disorder characterized by a diverse clinical presentation. Although rare, this has to be considered in multiorgan involvement when clinically appropriate. Cardiac disease is usually in the form of pericarditis, with rare myocardial involvement. Myocardial involvement can be either myocarditis or non-specific myocardial dysfunction in an intense inflammatory milieu. Treatment of myocarditis in AOSD has two arms, i.e., non-specific management of heart failure, and disease-specific treatment with corticosteroids, IL-1/IL-6 antagonists, or TNF inhibitors. Apart from disease flare, hepatic involvement is commonly due to autoimmune hepatitis, macrophage activation syndrome, portal vein thrombosis, primary biliary cirrhosis, hepatitis virus reactivation, fatty liver, and DILI. Long-term prognosis is favorable, with most of the patients having complete recovery of cardiac function on follow-up.

## References

[1] Efthimiou P, Kontzias A, Hur P, Rodha K, Ramakrishna GS, Nakasato P (2021). Adult-onset Still's disease in focus: clinical manifestations, diagnosis, treatment, and unmet needs in the era of targeted therapies. Semin Arthritis Rheum.

[2] Bodard Q, Langlois V, Guilpain P (2021). Cardiac involvement in adult-onset Still's disease: manifestations, treatments and outcomes in a retrospective study of 28 patients. J Autoimmun.

[3] Yamaguchi M, Ohta A, Tsunematsu T (1992). Preliminary criteria for classification of adult Still's disease. J Rheumatol.

[4] Henter JI, Horne A, Aricó M (2007). HLH-2004: diagnostic and therapeutic guidelines for hemophagocytic lymphohistiocytosis. Pediatr Blood Cancer.

[5] Bywaters EG (1971). Still's disease in the adult. Ann Rheum Dis.

[6] Gottschalk MN, Heiland M, Nahles S, Preissner R, Petri WA, Wendy S, Preissner S (2023). Increased incidence of adult-onset Still's disease in association with COVID-19 vaccination and SARS-CoV-2 infection. Orphanet J Rare Dis.

[7] Rao S, Tsang LS, Zhao M, Shi W, Lu Q (2022). Adult-onset Still's disease: a disease at the crossroad of innate immunity and autoimmunity. Front Med (Lausanne).

[8] Wang MY, Jia JC, Yang CD, Hu QY (2019). Pathogenesis, disease course, and prognosis of adult-onset Still's disease: an update and review. Chin Med J (Engl).

[9] Fautrel B, Zing E, Golmard JL (2002). Proposal for a new set of classification criteria for adult-onset still disease. Medicine (Baltimore).

[10] Sagar S, Liu PP, Cooper LT Jr (2012). Myocarditis. Lancet.

[11] Smith SC, Ladenson JH, Mason JW, Jaffe AS (1997). Elevations of cardiac troponin I associated with myocarditis. Experimental and clinical correlates. Circulation.

[12] Gerfaud-Valentin M, Sève P, Iwaz J (2014). Myocarditis in adult-onset still disease. Medicine (Baltimore).

[13] L'Heureux M, Sternberg M, Brath L, Turlington J, Kashiouris MG (2020). Sepsis-induced cardiomyopathy: a comprehensive review. Curr Cardiol Rep.

[14] Gracia-Ramos AE, Contreras-Ortíz JA (2020). Myocarditis in adult-onset Still's disease: case-based review. Clin Rheumatol.

[15] Giacomelli R, Ruscitti P, Shoenfeld Y (2018). A comprehensive review on adult onset Still's disease. J Autoimmun.

